# Supplementary material for: Transverse barrier formation by electrical triggering of a metal-to-insulator transition
Source: Nat Commun. 2021 Sep 17;12:5499. doi: 10.1038/s41467-021-25802-1 (PMC8448889; doi:10.1038/s41467-021-25802-1)
Supplement: Supplementary file 1 — Supplementary Information [file 41467_2021_25802_MOESM1_ESM.pdf]

Transverse barrier formation by electrical triggering of a metal-to-insulator transition

Pavel Salev[1], Lorenzo Fratino[2], Dayne Sasaki[3], Rani Berkoun[2], Javier del Valle[1,†], Yoav Kalcheim [1,‡], Yayoi Takamura[3], Marcelo Rozenberg[2], Ivan K. Schuller[1]*

[1]Department of Physics and Center for Advanced Nanoscience, University of California San Diego, La Jolla, California 92093, USA

[2]Laboratoire de Physique des Solides, CNRS, Université Paris-Sud, Université Paris-Saclay, 91405 Orsay Cedex, France

[3]Department of Materials Science and Engineering, University of California Davis, Davis, California 95616, USA

*Corresponding author: psalev@ucsd.edu

[†]Present address: Department of Quantum Matter Physics, University of Geneva, 24 Quai Ernest-Ansermet, 1211 Geneva, Switzerland

[‡] Present address: Department of Materials Science and Engineering, Technion-Israel Institute of Technology, 32000 Haifa, Israel

Supplementary Information 1

The structural quality of the synthesized $La_{0.7}Sr_{0.3}MnO_3$ (LSMO) films was examined using x-ray diffraction. Supplementary Fig. 1a shows a specular θ-2θ scan in the vicinity of the $SrTiO_3$ (STO) (002) Bragg peak. The LSMO $(002)_{pc}$ peak is at Q = 3.25 $Å^{-1}$ giving an out-of-plane lattice constant of 3.86 Å. The film's diffraction pattern has clear Laue oscillations, which attest to a coherent crystal structure with smooth interfaces. Supplementary Fig. 1b shows a reciprocal space map in the vicinity of the STO (103) peak. The LSMO film peak has the same Q_x component as the STO substrate, indicating that the film is fully strained.

Supplementary Fig. 1c shows the resistance-temperature dependence of the film before patterning (grey line) and of the fabricated devices (green and red). The three curves display the same metal-insulator transition (MIT) in terms of transition temperature and magnitude of the resistance change. This behavior implies that the device fabrication preserved the film's chemical and structural integrity.

Supplementary Information 2

We found that the volatile resistive switching in our LSMO device is due to the triggering of MIT mediated by Joule heating. We found no evidence that oxygen electromigration, which often produces nonvolatile resistive switching in binary and complex oxides, plays any significant role in our switching experiments.

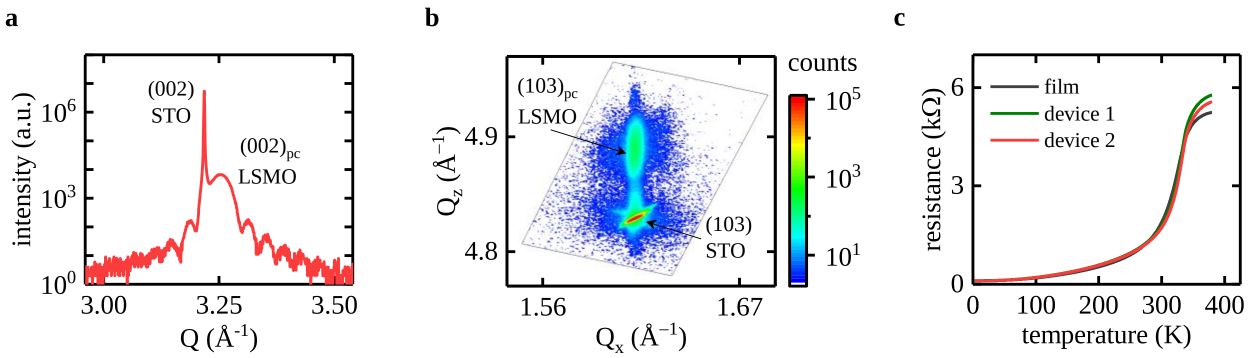

Supplementary Fig. 1. a, b, X-ray diffraction of the LSMO film: specular θ-2θ scan in the vicinity of the $SrTiO_3$ (002) peak (**a**) and reciprocal space map in the vicinity of the $SrTiO_3$ (103) peak (**b**). **c,** Resistance vs. temperature curves of the LSMO film (grey line) and two 50×100 μm^2 devices (green and red lines) showing similar behavior.

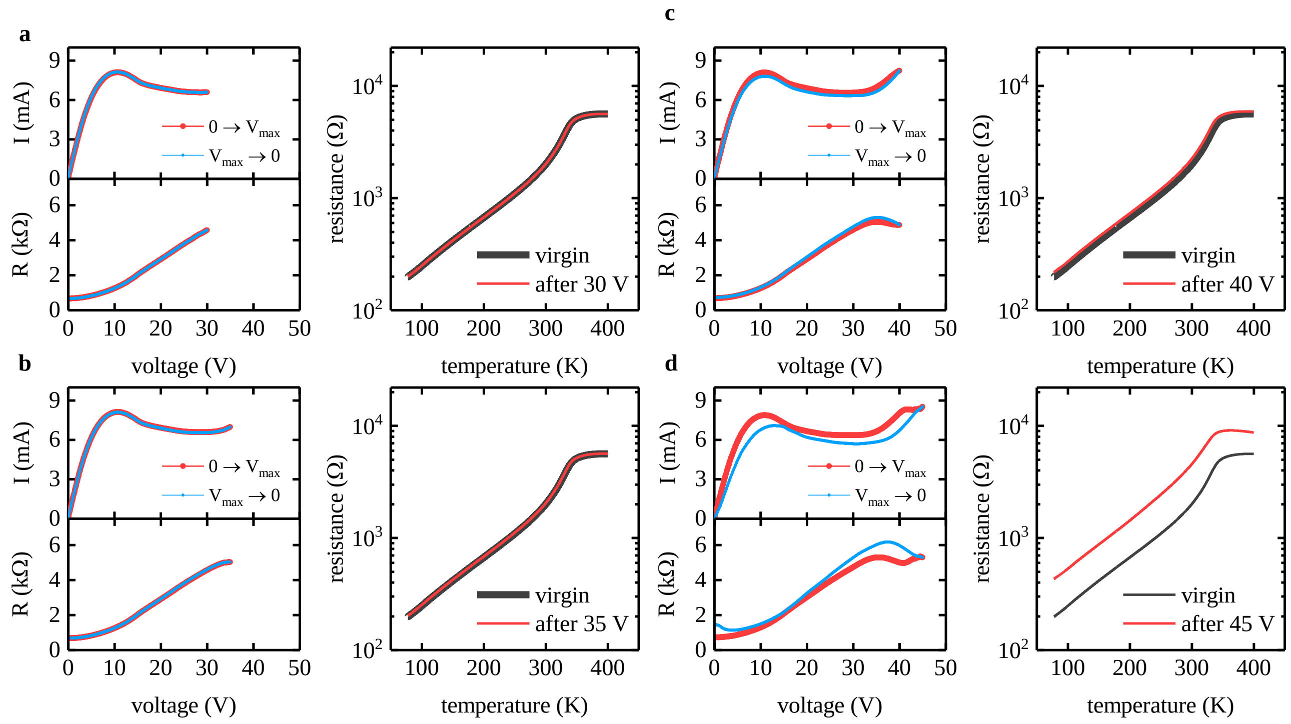

Supplementary Fig. 2.1. Performing resistive switching in LSMO has no impact on the resistance-temperature dependence (**a** and **b**) unless very high voltages are applied (**c** and **d**). In the I-V plots, the overlaid red and blue lines correspond to the increasing and decreasing voltage cycles, respectively. The I-V measurements were done at 200 K.

Below we list the evidence supporting the electro-thermal origin of the observed switching in LSMO:

1. Reproducible switching is present in every as-made LSMO device without the need of an electroforming. In contrast, electroforming is often necessary to initiate oxygen migration[1].

2. The switching is volatile, i.e. the device resets in the initial low-resistance state automatically upon turning off the driving voltage/current as expected for the Joule heating mediated process.

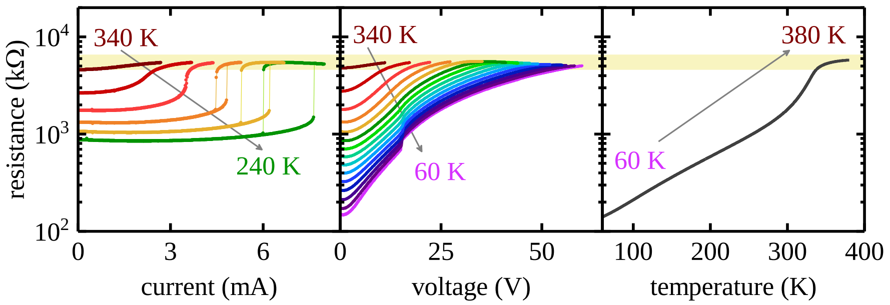

Supplementary Fig. 2.2. Comparison of the resistance change during current- (left) and voltage-controlled (middle) resistive switching to the equilibrium resistance-temperature dependence (right) of an LSMO device. The scale of the vertical axis (resistance) is the same in all three plots to facilitate the comparison.

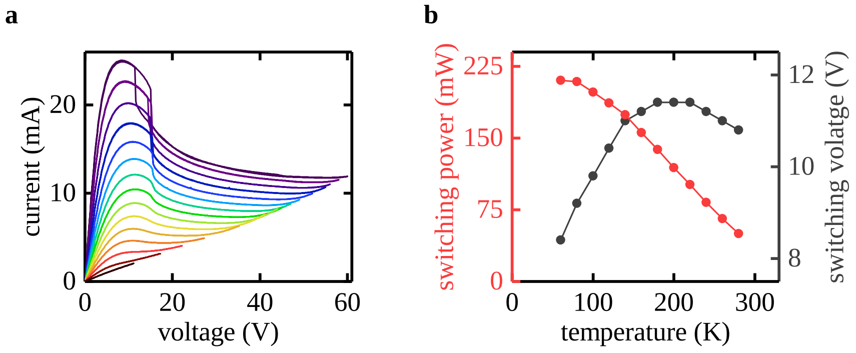

Supplementary Fig. 2.3. a, Voltage-controlled I-V curves recorded in a 60–340 K range using a step of 20 K. **b,** Switching power and voltage corresponding to the onset of the NDR region extracted from the I-V curves in **a**.

3. Performing the switching measurements has no impact on the resistance-temperature dependence as demonstrated in Supplementary Fig. 2.1. Because the switching is volatile and non-destructive, the resistance-temperature dependencies of the virgin device and of the device after the switching are identical (Supplementary Fig. 2.1 a and b). It is possible, however, to induce a permanent change by applying too large voltage (Supplementary Fig. 2.1 c and d). This permanent change of the resistance-temperature dependence could be due to the initiation of oxygen migration in the LSMO device and/or degradation of the electrode/oxide interface. During the acquisition of data presented in the main text and in the Supplementary Information, the application of high voltages/currents that could damage the device was avoided, unless stated otherwise. In this demonstration, the resistive switching measurements were performed 200 K.

4. The switching occurs in a wide temperature range up to $T_c \approx 340$ K where the strong I-V nonlinearities disappear, which indicates a close relation between the switching and the MIT (see Fig. 2 b and c in the main text and Supplementary Fig. 2.3).

5. The high resistance state of ~5.5 kΩ attained after the switching remains the same independent of the measurement temperature. This high-resistance state corresponds to the maximum resistance in the equilibrium resistance-temperature dependence as demonstrated in Supplementary Fig. 2.2. The resistance-current and resistance-voltage characteristics were calculated using the measured I-V data. The comparison in Supplementary Fig. 2.2 indicates that the resistive switching triggers the same MIT as occurs in the LSMO device by increasing temperature.

6. Resistive switching is accompanied by the coupled magnetic transition as discussed in the main text.

7. MIT and ferromagnetism in LSMO are extremely sensitive to stoichiometry, defects and disorder[2]. However, neither MIT nor ferromagnetism are affected by cycling the LSMO device back and forth

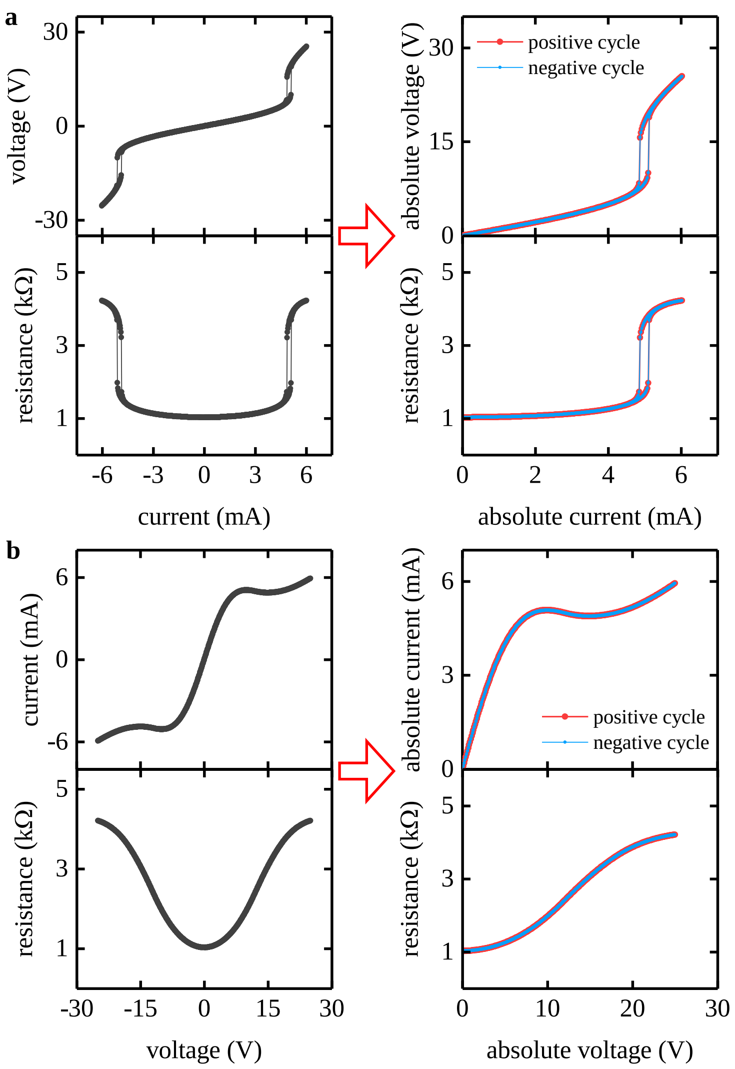

Supplementary Fig. 2.4. Current- (**a**) and voltage-controlled (**b**) I-V curves recorded using symmetric positive/negative driving stimulus. Taking the absolute values of current/voltage produces perfectly coinciding I-V curves of positive and negative cycles. The measurements were done at room temperature.

through the volatile resistive switching, even when the switching is induced in the entire 50×100 μm^2 device under the application of large voltages/currents (see Figs. 2 and 3 in the main text and Supplementary Fig. 3). If the ionic migration was responsible for the observed switching, it would be impossible that the stoichiometry and crystal structure could spontaneously restore to the pristine state within the 50×100 μm^2 device area when the voltage/current are turned off and no indication of chemical/structural change during the switching could be found in the MIT and ferromagnetic properties. The observed behavior, on the other hand, is consistent with the Joule heating origin of the switching. Warming up the device just past $T_c \approx 340$ K is not expected to cause material decomposition, thus neither MIT not ferromagnetic properties are affected by the switching.

8. The switching power steadily increases with decreasing temperature, as expected for the Joule heating mediated process. Supplementary Fig. 2.3a shows voltage-controlled I-V curves recorded in a 60–340 K temperature range. Several I-V curves from this figure are also shown in the main text in Fig. 1c. To characterize the switching parameters, we extracted the currents and voltages corresponding to the onset of negative differential resistance (NDR), i.e. the point at which dV/dI changes sign.

Supplementary Fig. 2.3b shows the switching power and the switching voltage dependence on temperature. While switching power has a monotonic temperature dependence, the switching voltage shows a non-monotonic behavior. This result suggests that Joule heating rather than electric field drives the metal-to-insulator switching in our LSMO devices.

9. The switching is perfectly symmetric with respect to changing the driving voltage/current polarity (i.e. a unipolar switching). As shown in Supplementary Fig. 2.4, the I-V curves recorded using positive and negative driving stimuli perfectly coincide with each other when plotted on the absolute current/voltage scale. This is the expected behavior for the Joule heating mediated process because the electric power dissipated in the device is proportional to the square of driving current/voltage, $P = I^2R = V^2/R$. In this demonstration, the resistive switching measurements were performed at room temperature.

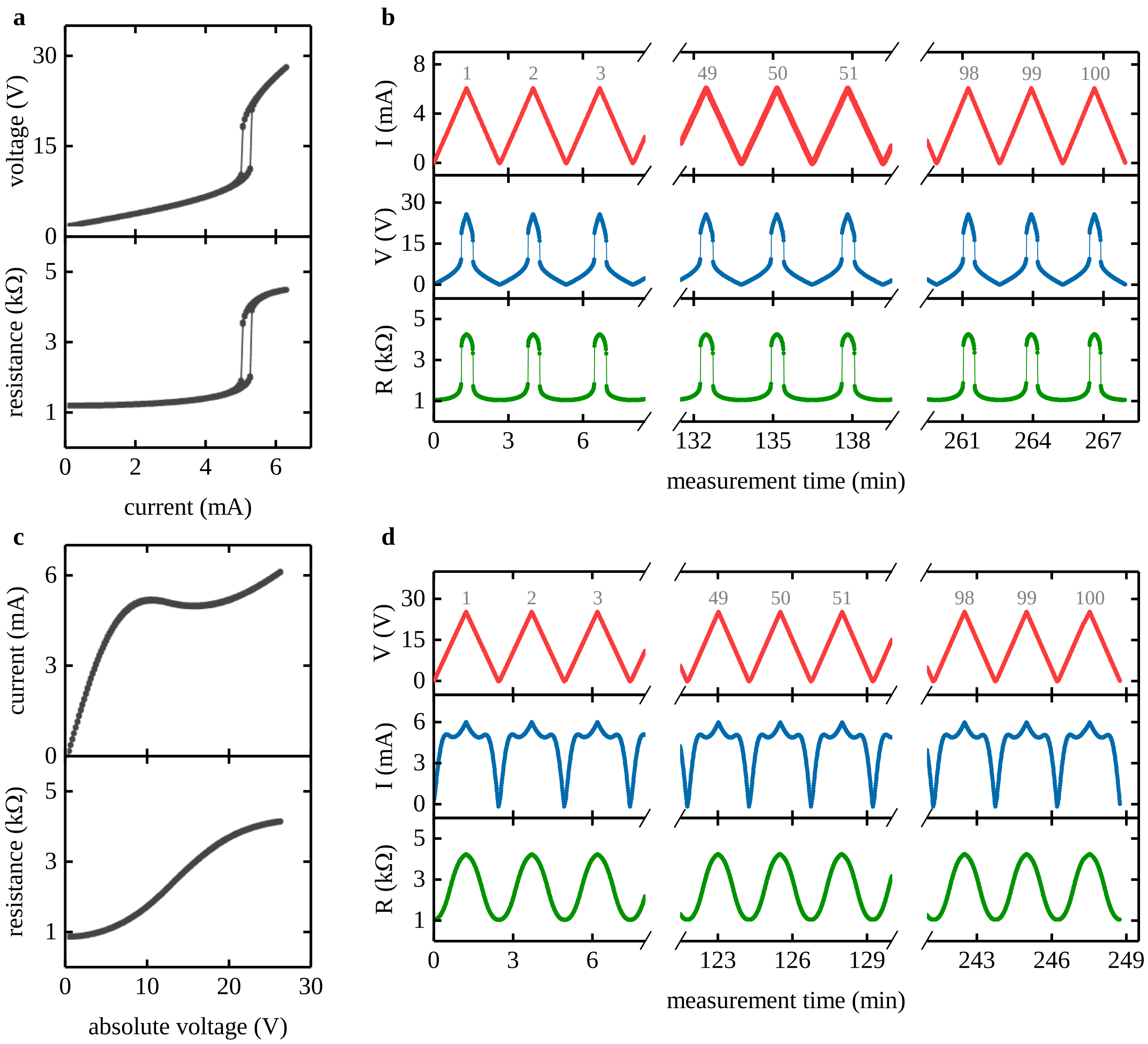

Supplementary Fig. 2.5. Current- (**a** and **b**) and voltage-controlled (**c** and **d**) resistive switching cycling tests. The I-V curves in panels **a** and **c** show **100 overlaid cycles**, which perfectly coincide with one another. Panels **b** and **d** show the zoomed-in time traces of the cycling measurements, which demonstrate that switching behavior does not change from the first to the last cycle. The gray numbers indicate the cycle numbers. The cycling tests were done at room temperature.

10. The switching is repeatable over a large number of cycles having virtually no cycle-to-cycle variations of low- and high-resistance states, of switching voltages and currents, of the shape of the I-V curves, etc. Supplementary Fig. 2.5 shows the effect of performing 100 consecutive current- and voltage-controlled switching cycles. The switching cycles were performed in a dc-like mode resulting in over 8 hours of high voltage/current treatment of the sample. The overlaid 100 I-V curves in Supplementary Fig. 2.5 a and c perfectly coincide with each other. The absence of cycle-to-cycle variability in the volatile MIT-based switching is in the stark contrast with typical high variability in nonvolatile oxygen-migration based switching[3]. When Joule heating triggers the electronic MIT in LSMO, the chemical and structural integrity of the material is preserved. The LSMO device remains exactly the

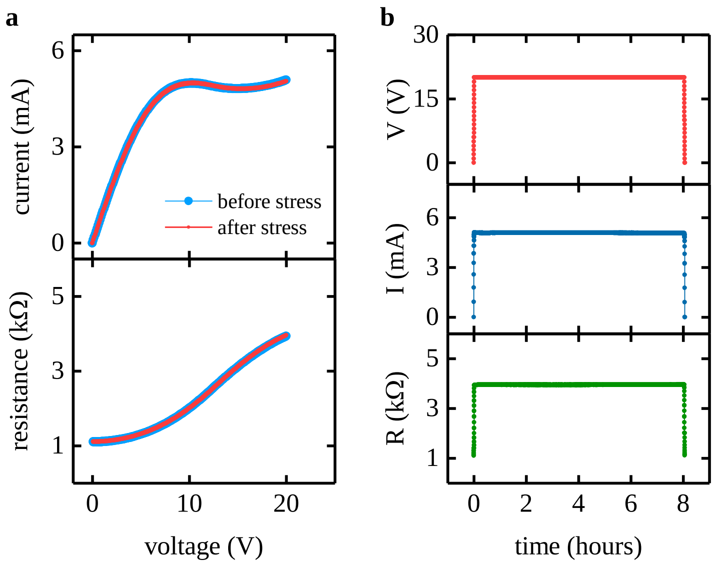

Supplementary Fig. 2.6. a, I-V (top) and R-V (bottom) curves recorded before (blue line) and after (red line) 8-hour long 20 V stress test. **b,** Voltage, current and resistance time traces recorded during the stress test. The long stress test has no impact on the volatile MIT-based resistive switching in LSMO. All measurements were done at room temperature.

same after each switching cycle, which results in the absence of cycle-to-cycle variability. The cycling tests were performed at room temperature.

11. Under constant applied voltage, the high-resistance state can be maintained persistently for prolonged time without any signs of device degradation. In contrast, continual resistance change under constant voltage is often observed in memristors based on oxygen migration[4–8]. Supplementary Fig. 2.6 shows an 8-hour long voltage stress test. The measurements were done using 20 V stress, which is high enough to induce and maintain the high-resistance state at room temperature. While the applied voltage was kept on, we observed no resistance drift over the entire duration of the experiment. The I-V curves before and after the test perfectly coincide with each other indicating that no nonvolatile switching or irreversible damage were induced by holding large stress voltage over a long time. The stress test was done at room temperature.

12. The switching in LSMO shows high endurance and no cycle-to-cycle variability over 5×10^6 high-speed switching cycles. The electrical circuit used in the high-speed measurements is shown in Fig. 2.7a. Because of the large size devices optimized for MOKE imaging (50×100 μm^2), we had to use a combination of a function generator and an amplifier to produce large enough voltage/current to induce the switching of the entire device. The performance of the high-voltage amplifier determined the limit of how fast a switching cycle can be performed (15-ms-period waveform) and ultimately set the limit of how many switching cycles (5×10^6) can be acquired in a reasonable amount of time. Reducing the device size down to nanoscale dimensions should reduce the switching voltage/current enabling fast speed measurements to determine the ultimate switching time in LSMO and to probe the device endurance past several million cycles. Fig. 2.7b shows the dynamic I-V curves measured in a 50×100 μm^2 LSMO device. The dynamic I-V curves have similar appearance to the dc I-V curves (for example in Supplementary Fig. 2.4a). The larger hysteresis in the dynamic I-V curves is consistent with the Joule heating origin of the switching as thermal equilibrium in a large-size device cannot be established quickly. All the I-V curves recorded between the 1 and 5×10^6 cycles are identical resulting in no apparent dependence of the low- and high-resistance states on cycling (Supplementary Fig. 2.7c).

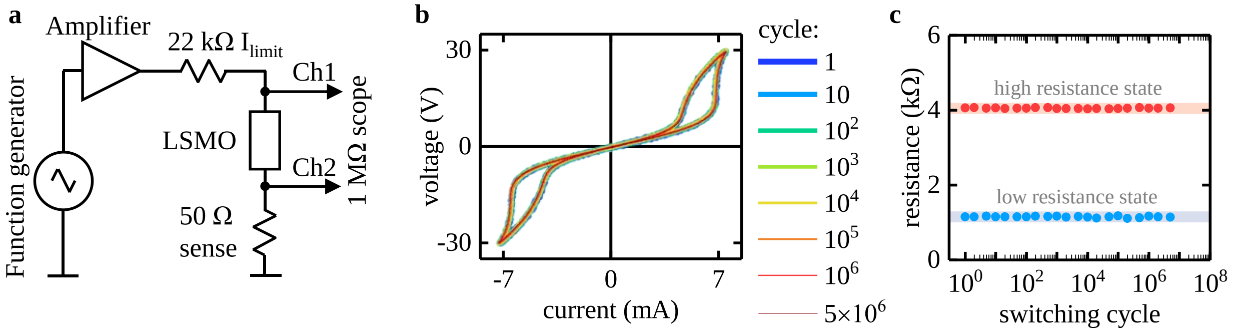

Supplementary Fig. 2.7. Fast switching cycling test. **a**, The measurement circuit that was used to apply a triangular 15-ms-period waveform to probe the switching in the LSMO device. **b**, Overlaid dynamic I-V curves recorded by an oscilloscope for different consecutive switching cycles ranging from cycle 1 to cycle 5×10^6 . No change in the I-V shape can be observed. **c**, Low- and high-resistance values extracted from the I-V curves as functions of switching cycles. The two resistance states are well separated and remained unaffected by 5×10^6 switching cycles.

The statistical analysis of the low- and high-resistance states over the switching cycles gives R_{low} = 1150 ± 16 Ω and R_{high} = 4050 ± 10 Ω. The observed resistance cycle-to-cycle deviations (16 Ω and 10 Ω) are well within the accuracy of the "single-shot" oscilloscope measurements, indicating the absence of the device degradation and extremely high repeatability of the volatile resistive switching in LSMO. While high-endurance switching can be achieved in systems based on ionic migration, the lack of variability in the I-V shape or the low- and high-resistance states over 5×10^6 cycles suggests that the observed volatile switching in LSMO is not related to ionic migration.

13. The switching is independent of the oxygen partial pressure. Supplementary Fig. 2.8 compares current- and voltage controlled I-V curves recorded in air and in high vacuum (~10^{-7} Torr). The switching behavior in both cases is identical, which indicates that the oxygen partial pressure does not play a significant role in the volatile resistive switching in LSMO. On the contrary, the nonvolatile resistive switching in LSMO driven by oxygen migration has strong dependence on the environmental oxygen pressure[9]. In Supplementary Fig. 2.7, small deviations in the I-V curves recorded in air and in vacuum most likely are due to a small modification of thermal conditions, which are expected to play a significant role in the Joule heating mediated switching process. In vacuum, the LSMO sample exchanges heat with the sample stage. In air, the sample can exchange heat both with the sample stage and with air. The switching measurements were performed at room temperature.

14. It is possible to induce nonvolatile resistive switching in our devices by first applying a large voltage (i.e. performing electroforming) and then cycling the device in moderate voltages (Supplementary Fig. 2.9). During the electroforming, an irreversible breakdown occurs at ~45 V. After the breakdown, a hysteretic I-V curve showing a consistent nonvolatile switching can be obtained by cycling the device in ±6 V. This voltage is much lower, about factor of 5, compared to the voltage required to induce the MIT-based volatile switching, which could be an indication that the nonvolatile switching occurs in a small volume within the device, while the volatile switching happens throughout the entire device (as we established using MOKE measurements). The nonvolatile switching occurs between R_{low} ~ 230 Ω and R_{high} ~ 440 Ω giving the resistance switching ratio of ΔR/R ~ 90%. We note that both R_{high} and R_{low} states after the electroforming are noticeably different from the original resistance of ~660 Ω

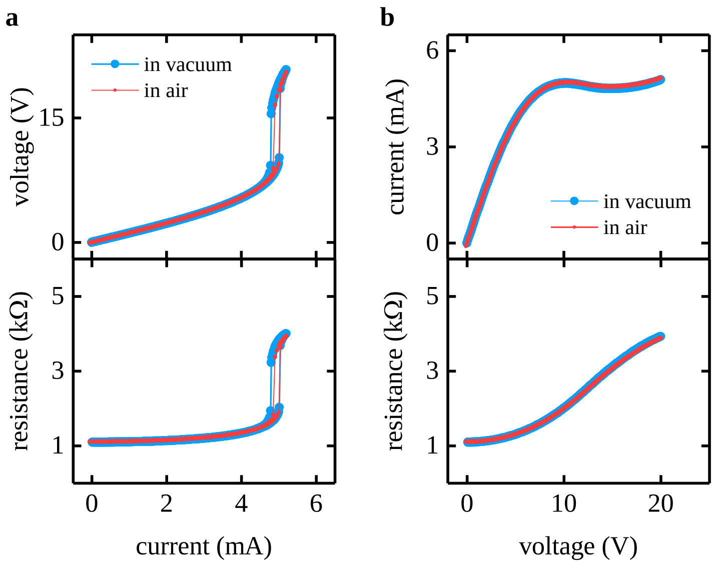

Supplementary Fig. 2.8. Current- (**a**) and voltage-controlled (**b**) resistive switching measurements in high vacuum (~10^{-7} Torr, blue line) and in air (red line). Oxygen partial pressure has no significant impact on the volatile MIT-based resistive switching in LSMO. The measurements were done at room temperature.

before the electroforming. In addition, the resistance-temperature dependence before and after the electroforming are dramatically different. The above observations indicate that the electroforming induced chemical and/or structural change inside the LSMO device. Overall, the I-V curves recoded during the nonvolatile switching have different shape and display a noticeably large cycle-to-cycle variability as compared to the volatile MIT-based switching, which highlights the different physical origin between the nonvolatile and volatile resistive switching in LSMO. Inducing a nonvolatile switching in LSMO by applying a high voltage is a qualitatively similar phenomenon as found in VO_2 and V_2O_3, where a small voltages/currents cause a volatile insulator-to-metal switching, but a large stimulus triggers the nonvolatile oxygen migration[10].

While several of the above properties could be sometimes found in resistive switching systems based on ionic migration, the fact that **all** of those properties are present **at the same time** provide strong evidence that the origin of the volatile resistive switching in LSMO devices is the triggering of MIT mediated by Joule heating.

We note that inducing the volatile resistive switching in our LSMO devices required the currents ranging from ~4 mA near room temperature to ~25 mA at 60 K. For a 50×100×0.02 μm^3 device, those current corresponds to the current density of 0.4-2.5×10^6 A/cm^2. The current densities used in our work are considerably larger compared to the previous work that reported the absence of Joule heating mediated switching in LSMO in 10^4-10^5 A/cm^2 range[11].

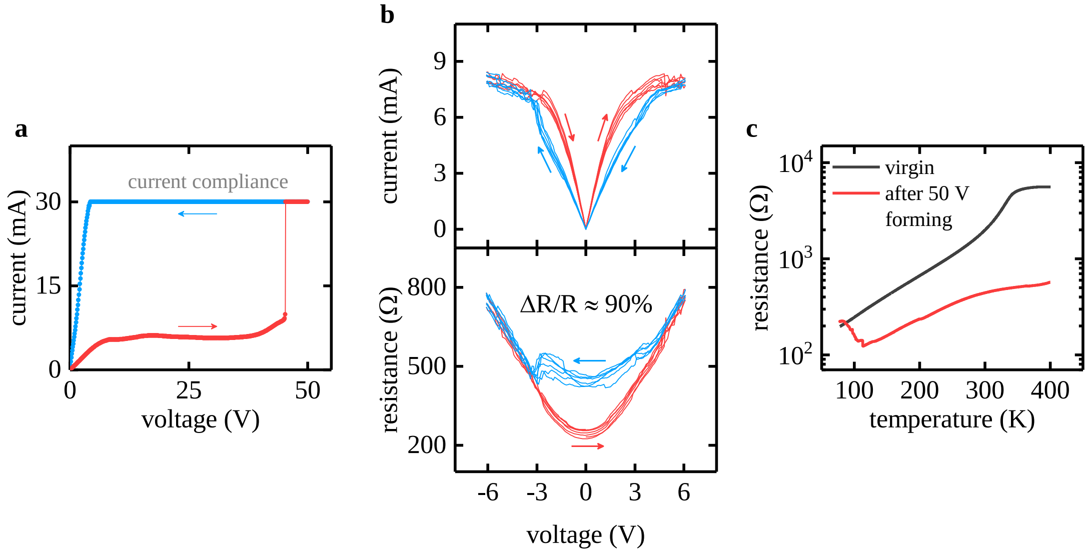

Supplementary Fig. 2.9. Nonvolatile resistive switching in LSMO device. **a,** Initial 50 V electroforming cycle showing an irreversible breakdown at ~45 V. **b,** Voltage-controlled I-V curves recorded after the electroforming. Nonvolatile switching of ΔR/R ~ 90% can be observed. Six consecutive switching cycles are shown. Unlike the volatile MIT-based switching (see Supplementary Fig. 2.5), the nonvolatile switching displays noticeable cycle-to-cycle variability, which is common behavior in resistive switching systems based on oxygen migration. **c,** Comparison of the resistance-temperature dependence before and after the electroforming. Unlike the volatile MIT-based switching (see Supplementary Fig. 2.3), the electroforming (which is necessary to initiate the nonvolatile switching in our devices) dramatically changes the resistance-temperature dependence. The electroforming and nonvolatile switching measurements were done at 200 K.

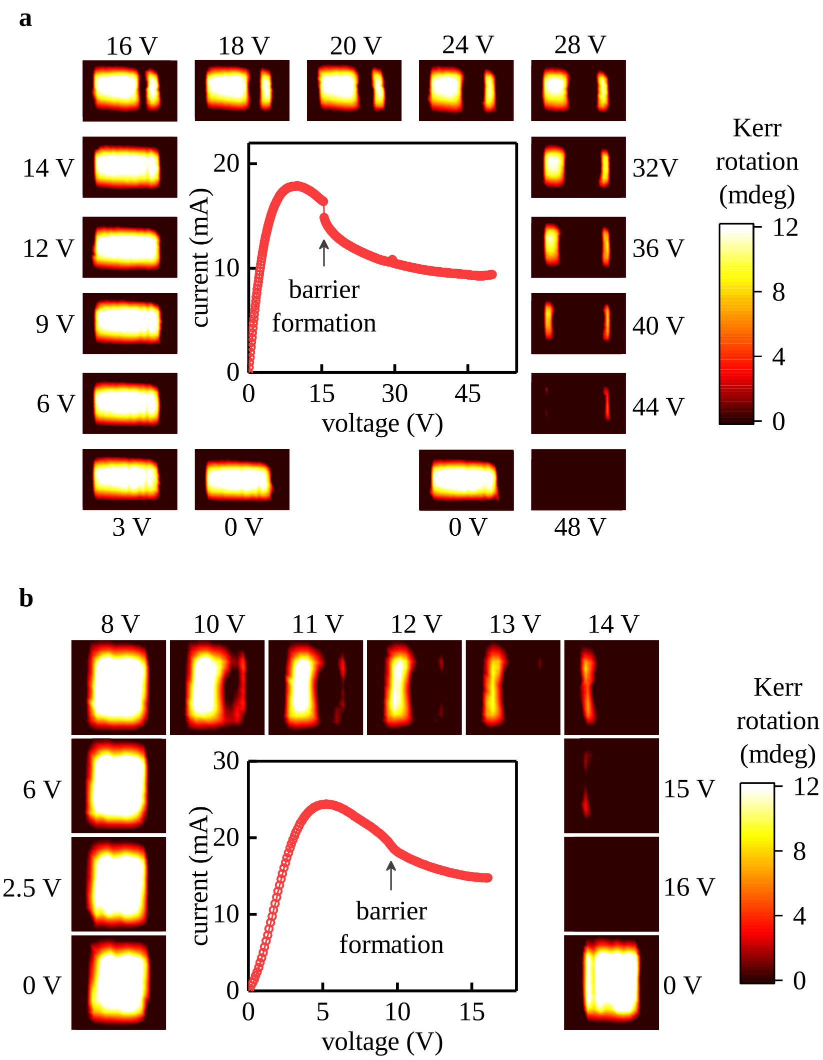

Supplementary Fig. 3. a, b, Simultaneously recorded I-V curve (center) and MOKE amplitude *xy*-maps (sides) in two different samples having different device geometry. Device in **a** is patterned in a 20 nm thick film and has 50×100 μm^2 size. Device in **b** is patterned in a 50 nm thick film and has 50×50 μm^2 size. The field of view in the MOKE maps is 90×140 μm^2 in **a** and 75×75 μm^2 in **b**. In the maps, the current flows horizontally. the All measurements were performed at 100 K.

Supplementary Information 3

Supplementary Fig. 3 shows the MOKE maps at different voltages and the corresponding I-V curves recorded in the same sample as in the main text but in a different device (panel a) and in another LSMO sample of different film thickness (50 nm) and different device dimensions (50×50 μm^2, panel b). In both cases, we observed the same behavior as described in the main text: the switching from a metal into an insulator occurs by the formation of an insulating barrier that spans through the entire device width in the direction perpendicular to the current flow. The repeatability of the switching behavior indicates that the formation of an insulating barrier is a general property of the metal-to-insulator switching.

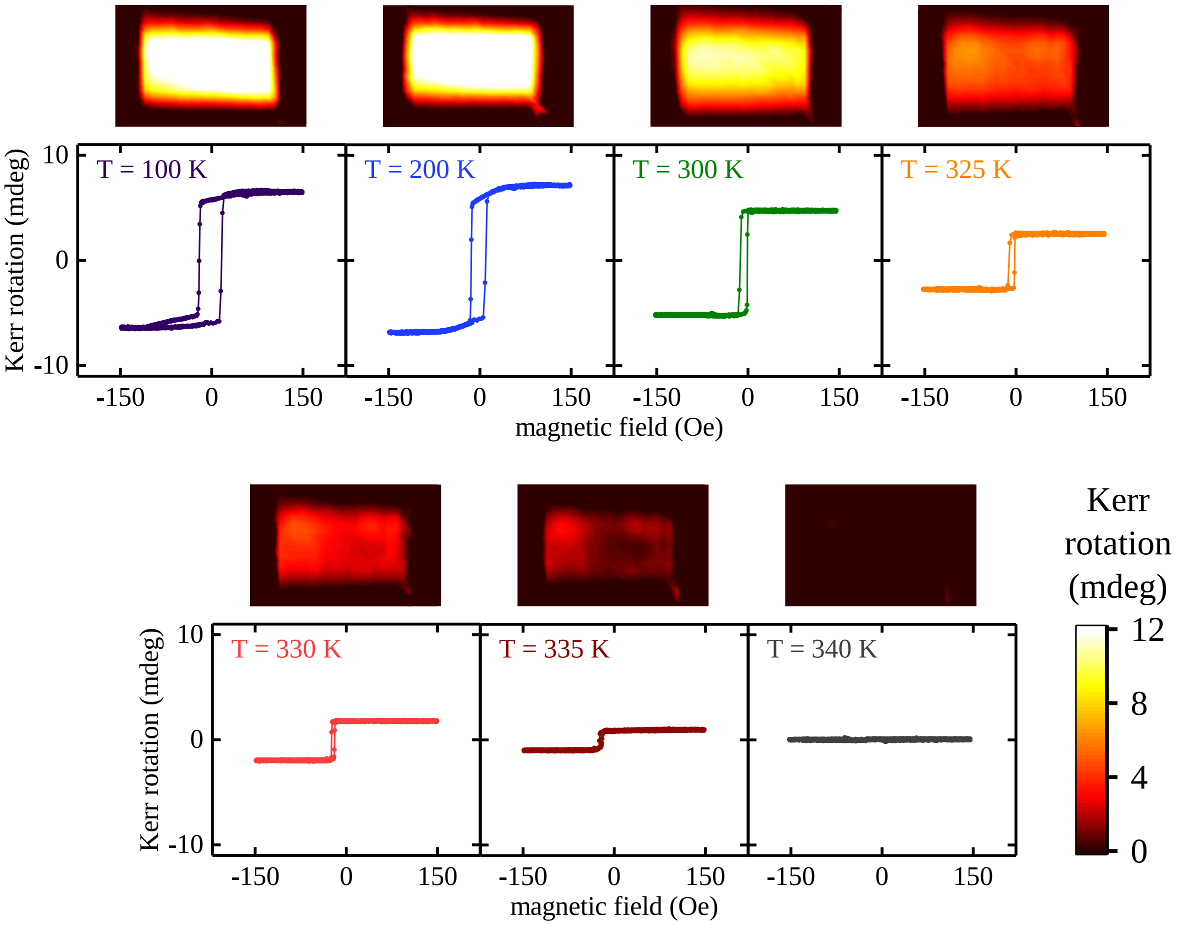

Supplementary Fig. 4. MOKE maps and corresponding MOKE hysteresis loops at different temperatures recorded during the equilibrium thermal transition (i.e. without applying voltage/current to the LSMO device). In contrast with the formation of an insulating barrier, the thermal transition is spatially uniform. The field of view in the MOKE maps is 90×140 μm^2.

Supplementary Information 4

To check whether the formation of an insulating barrier is not just an anomaly of the LSMO film, which, for example, could be introduced during the device fabrication, we measured the MOKE maps over 100–400 K temperature range without applying voltage (Supplementary Fig. 4). We used the same imaging procedure and the same magnetic field settings as described in Methods section in the main text. Under equilibrium conditions, we observed a spatially uniform transition throughout the device. Importantly, we found no correlation to the formation of an insulating barrier that we observed during resistive switching. This result demonstrate that the formation of an insulating barrier is a special property of the electrically-driven metal-to-insulator transition.

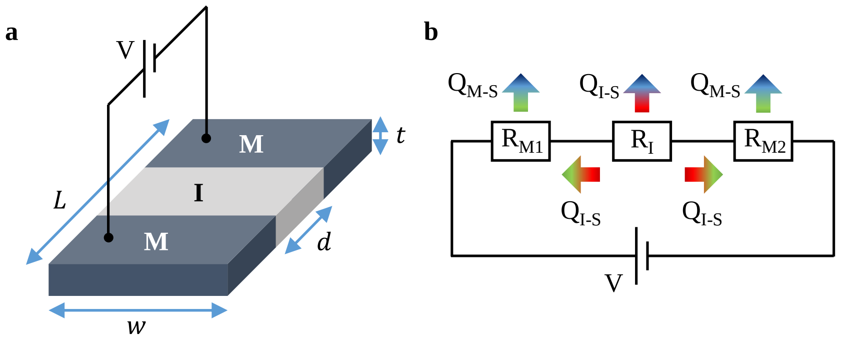

Supplementary Fig. 5.1. a, Device geometry considered in the analytical model: an insulating barrier in the middle separates the two metallic parts. Device dimensions used in the equations are shown. **b,** Schematic of the analytical model showing the electrical circuit and heat exchange flows.

Supplementary Information 5

To derive an analytical equation for the insulating barrier size we consider a simple model consisting of series resistors that represent metal/insulator/metal configuration and we take into account the heat exchange between the metal regions, insulator region, and the substrate (Supplementary Fig. 5.1). At equilibrium, i.e. $\partial T/\partial t = 0$, the heat equation for the insulating barrier can be written as

$$\frac{V_{apl}^2 R_I}{(R_M + R_I)^2} = k_s(T_I - T_0) + 2k_f(T_I - T_M) \quad (5.1)$$

where R_I and R_M are resistances of insulator and metal, T_I, T_M, and T_0 are temperatures of insulator, metal, and substrate, k_f and k_s are thermal conductivities of heat exchange within the film and between the film and the substrate. Introducing material properties and device dimensions as (defined in Supplementary Fig. 5.1 a)

$$\begin{aligned} R_I &= \rho_I \frac{d}{t \cdot w} \\ R_M &= \rho_M \frac{L-d}{t \cdot w} \\ k_s &= \kappa_s (d \cdot w) \\ k_f &= \kappa_f (t \cdot w) \end{aligned} \quad (5.2)$$

equation (5.1) can be rewritten in a compact form

$$\boldsymbol{A}\left(\frac{d}{L}\right)^3 + \boldsymbol{B}\left(\frac{d}{L}\right)^2 + \boldsymbol{C}\left(\frac{d}{L}\right) + \boldsymbol{D} = 0 \quad (5.3)$$

where

$$\begin{aligned} \boldsymbol{A} &= \kappa_s \alpha^2 \\ \boldsymbol{B} &= 2\kappa_s \alpha + 2\kappa_f \alpha^2 \frac{t}{L}\frac{(T_I - T_M)}{(T_I - T_0)} \\ \boldsymbol{C} &= \kappa_s + 4\kappa_f \alpha \frac{t}{L}\frac{(T_I - T_M)}{(T_I - T_0)} - \frac{(\alpha+1)t}{\rho_M (T_I - T_0) L^2} V_{apl}^2 \end{aligned} \quad (5.4)$$

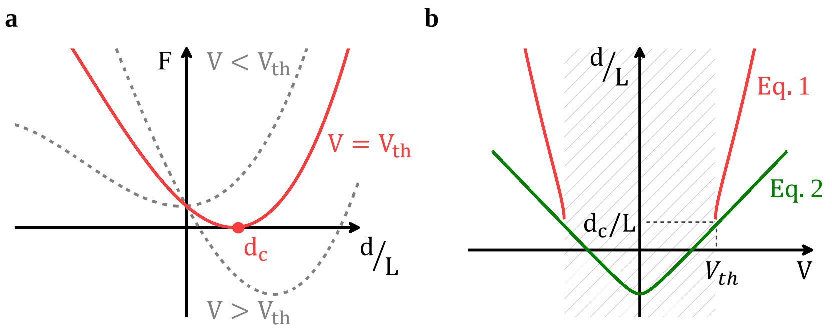

Supplementary Fig. 5.2. a, Schematic plot of equation (5.3) for three voltages: below and above the threshold (grey dashed lines) and at the threshold (red line). The minimum size of the insulating barrier d_c is highlighted. **b,** Schematic plot of system of equations (5.5). The intercept of the two curves corresponds to the minimum insulating barrier size d_c and threshold voltage V_{th} to induce such a barrier. The shaded area highlights the region where Eq. 1 becomes imaginary.

$$\boldsymbol{D} = 2\kappa_f \frac{t}{L}\frac{(T_I - T_M)}{(T_I - T_0)}$$

$$\alpha \equiv \frac{\rho_I - \rho_M}{\rho_M}$$

As can be seen from (5.4), all coefficients in (5.3) are positive when V_{apl} is small, therefore there is no positive solution for barrier size, d, i.e. the insulating barrier cannot form without the application of a strong enough voltage. We define the V_{th} as the threshold voltage at which (5.3) has the smallest positive solution d_c for the insulating barrier size. Supplementary Fig. 5.2a shows that at V_{th} the equation (5.3) has a local minimum corresponding to d_c. This means that the derivative of (5.3) is also zero at d_c, which leads to a system of two equations

$$\begin{cases} \frac{d_c}{L} = \frac{-\boldsymbol{C} + \sqrt{\boldsymbol{C}^2 - 4\boldsymbol{BD}}}{2\boldsymbol{B}} \\ \frac{d_c}{L} = \frac{-2\boldsymbol{B} + \sqrt{4\boldsymbol{B}^2 - 12\boldsymbol{AC}}}{6\boldsymbol{A}} \end{cases} \tag{5.5}$$

The above equations were derived assuming $d_c \ll L$ in order to obtain analytically solvable equations by dropping $(d_c/L)^3$ terms. Because of the missing cubic terms, system (5.5) actually does not have an exact solution. Supplementary Fig. 5.2b shows graphically that close to the intercept point, the first equation in (5.5) does not have a real value because the $\sqrt{\boldsymbol{C}^2 - 4\boldsymbol{BD}}$ term becomes imaginary. However, the two curves in Supplementary Fig. 5.2b come very close to the interception point. Therefore, we can write a condition for an approximate solution as following

$$\begin{cases} \frac{d_c}{L} = -\frac{\boldsymbol{C}}{2\boldsymbol{B}} \\ \boldsymbol{C}^2 - 4\boldsymbol{BD} = \boldsymbol{0} \end{cases} \tag{5.6}$$

System of equations (5.6) contains four unknowns: d_c, V_{th}, T_I, and T_M. In order to obtain an approximate solution, we used T_c and T_0 as estimations for the insulator and metal regions temperatures, T_I and T_M. As we found in our numerical simulations (see Fig. 3 in the main text), such estimations are justified. In addition, the temperature coefficients in (5.4), $(T_I - T_0)$ and $(T_I - T_M)$, are always multiplied by other parameters, such as α, κ_f, ρ_M. It is possible to "absorb" the modest deviations of $(T_I - T_0)$ and $(T_I - T_M)$ from $(T_c - T_0)$ into other parameters. Therefore, using $T_I \approx T_c$ and $T_M \approx T_0$ does fundamentally alter the

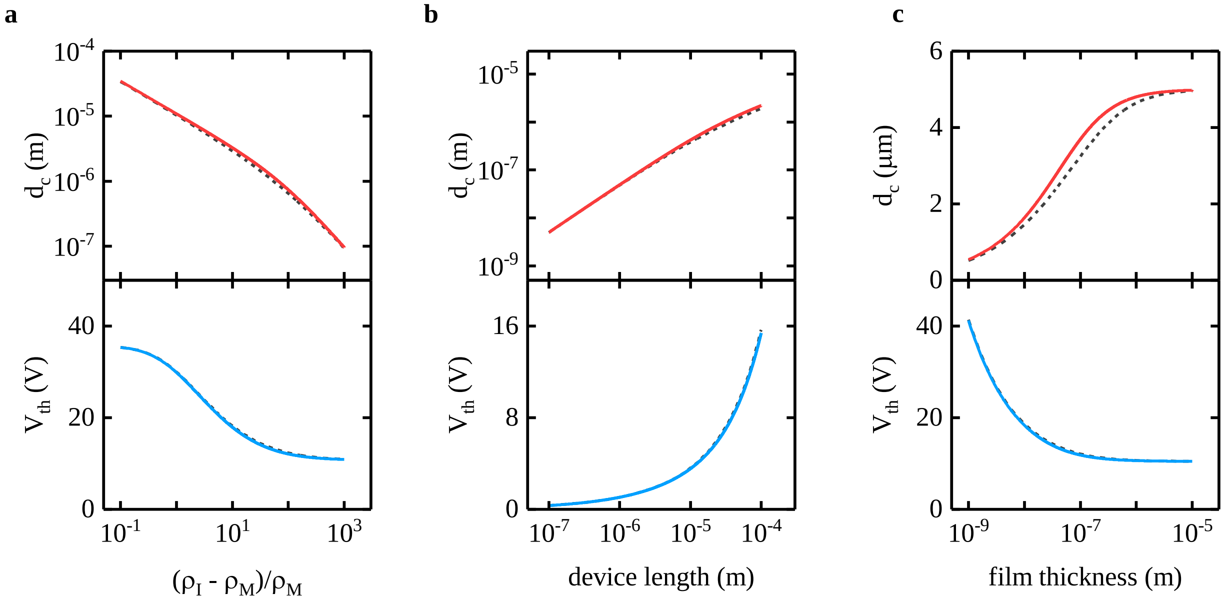

Supplementary Fig. 5.3. Minimum insulating barrier size (top graphs) and threshold voltage (bottom graphs) dependence on resistivity ratio (**a**), device length (**b**) and film thickness (**c**). Continuous red and blue curves were obtained using equations (5.7-5.8). Dashed gray lines were obtained by solving numerically equation (5.3). Material and device parameters were set to the values presented in the text (p. 7), with the exception of the specific parameter for which d_c and V_{th} were calculated (i.e. resistivity ratio (**a**), device length (**b**) and film thickness (**c**)).

physics of the problem, but greatly simplifies the analytical solution. System of equations (5.6) leads to the following expressions for the insulating barrier size d_c and the threshold voltage V_{th}

$$d_c \approx L\sqrt{\frac{1}{\alpha\left(\alpha + {}^{k_s L}\!/_{k_f t}\right)}} \tag{5.7}$$

$$V_{th} \approx \sqrt{\frac{k_f L \rho_M (T_c - T_0)}{\alpha + 1}\left(\frac{k_s L}{k_f t} + 4\left(\alpha + \sqrt{\alpha^2 + \alpha\frac{k_s L}{k_f t}}\right)\right)} \tag{5.8}$$

Supplementary Fig. 5.3 shows the dependence of the minimum barrier size and threshold voltage, d_c and V_{th}, on the resistivity ratio, device length, and film thickness. We plot the curves given by the approximate equations (5.7-5.8) (red and blue lines) and by numerically solving equation (5.3) using the $T_I \approx T_c$ and $T_M \approx T_0$ temperature estimations (grey dashed lines) and using the condition that the derivative of (5.3) is zero at V_{th} (see the discussion on p.6). The material and device parameters were $T_c = 340$ K, $T_0 = 100$ K, $\rho_M = 2\times10^{-6}$ Ω·cm, $\alpha = 20$, $L = 100$ µm, $t = 20$ nm, $k_s = 5\times10^6$ W·K^{-1}·m^{-2}, $k_f = 3\times10^8$ W·K^{-1}·m^{-2}. These parameters give $d_c = 2.2$ µm and $V_{th} = 15.4$ V, which is very close to the experimentally observed values. The two approaches give almost the same results, which further supports the validity of the approximation introduced in (5.6). The analytical model predicts that the minimum insulating barrier size can be substantially reduced, potentially down to nanoscale, by selecting a material with large

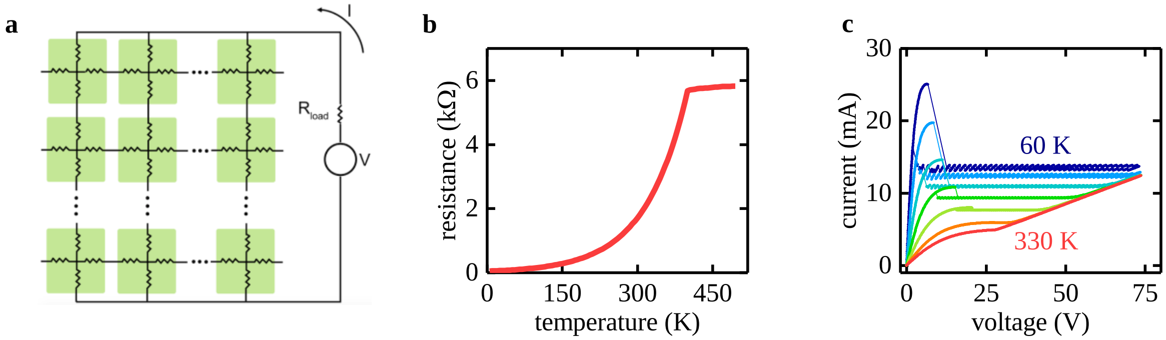

Supplementary Fig. 6. a, A schematic of the resistor network used in simulations of the metal-to-insulator resistive switching. Resistor values at each node depend on local temperature given by equations (6.1-6.2). **b,** Simulated resistance-temperature dependence of the resistor network. **c,** Simulated voltage-controlled I-V curves in 60 – 330 K temperature range.

insulator/metal resistivity ratio or by reducing the device dimensions, length and film thickness. We note that because of the simplifications made in our model these results should be regarded as guidelines rather than exact predictions.

Supplementary Information 6

The computational analysis of metal-to-insulator resistive switching was based on a resistor network model as shown schematically in Supplementary Fig. 6a. Each site of the 50×100 grid is represented by a 4-resistor node. The resistance of individual elements inside the nodes depends on temperature as

$$R(T) = \frac{R_0 e^{\alpha}}{m} + \frac{m-1}{m}\frac{R_0 e^{\alpha}}{1+e^{-\lambda(T-T_c)}} \tag{6.1}$$

where $R_0 = 18.5$, $m = 3$, $\lambda = 1.2 \times 10^{-4}$, $T_c = 340$, and

$$\alpha = \begin{cases} 0.012 \cdot T, & T < 400 \\ 0.012 \cdot 400, & T \geq 400 \end{cases} \tag{6.2}$$

These parameters were chosen to provide a semi-quantitative fit of the experimental $R(T)$ (see Fig. 1a in the main text). Supplementary Fig. 6b shows the $R(T)$ plot of the full resistor network.

I-V curves and resistance maps were calculated in an iterative way. For a given applied voltage, the resistor network is solved to obtain local voltages V_{ij} at each (i,j) site. Then these voltages are used to update local temperatures T_{ij} following the thermal diffusion equation:

$$\frac{dT_{ij}}{dt} = \frac{V_{ij}^2}{C_V R_{ij}} - \frac{k_h}{C_V}\left(5T_{ij} - T_{substrate} - \sum_{<kl>}^{1st\ neighboors} T_{kl}\right) \tag{6.3}$$

where $C_V = 2.0$ and k_h=0.4. Using the local temperatures, local resistances are updated according to equations (6.1-6.2). The iterative process of solving the resistor network and updating the local temperatures and local resistances is repeated until a steady state is found.

Supplementary Fig. 6c shows voltage-controlled I-V curves calculated in 60 – 330 K temperature range. Similar to the experimental data (se Fig. 1c in the main text and Supplementary Fig. 2a), we observed a strongly nonlinear behavior and the appearance of an N-type NDR region at the temperatures below 300 K. To better illustrate how the nonlinearities in the I-V curves are related to the formation of an insulating barrier, we created several animations of the simulation results, which can be accessed at [LINK].

1. Joshua Yang, J. *et al.* The mechanism of electroforming of metal oxide memristive switches. *Nanotechnology* **20**, 215201 (2009).
2. Tokura, Y. Critical features of colossal magnetoresistive manganites. *Reports Prog. Phys.* **69**, 797 (2006).
3. Ambrogio, S. *et al.* Statistical Fluctuations in HfO_x Resistive-Switching Memory: Part I - Set/Reset Variability. *IEEE Trans. Electron Devices* **61**, 2912 (2014).
4. Liu, Q. *et al.* Real-Time Observation on Dynamic Growth/Dissolution of Conductive Filaments in Oxide-Electrolyte-Based ReRAM. *Adv. Mater.* **24**, 1844 (2012).
5. Marinković, S. *et al.* Direct Visualization of Current-Stimulated Oxygen Migration in $YBa_2Cu_3O_{7-\delta}$ Thin Films. *ACS Nano* **14**, 11765–11774 (2020).
6. Wang, Z. *et al.* Memristors with diffusive dynamics as synaptic emulators for neuromorphic computing. *Nat. Mater.* **16**, 101 (2017).
7. Wu, X. *et al.* Intrinsic nanofilamentation in resistive switching. *J. Appl. Phys.* **113**, 114503 (2013).
8. Yang, Y. *et al.* Observation of conducting filament growth in nanoscale resistive memories. *Nat. Commun.* **3**, 732 (2012).
9. Gonzalez-Rosillo, J. C. *et al.* Engineering Oxygen Migration for Homogeneous Volume Resistive Switching in 3-Terminal Devices. *Adv. Electron. Mater.* **5**, 1800629 (2019).
10. del Valle, J. *et al.* Electrically Induced Multiple Metal-Insulator Transitions in Oxide Nanodevices. *Phys. Rev. Appl.* **8**, 054041 (2017).
11. Moreno, C. *et al.* Absence of self-heated bistable resistivity in $La_{0.7}Sr_{0.3}MnO_3$ films up to high current densities. *Phys. Rev. B* **80**, 094412 (2009).
