# Supplementary material for: Transverse barrier formation by electrical triggering of a metal-to-insulator transition
Source: Nat Commun. 2021 Sep 17;12:5499. doi: 10.1038/s41467-021-25802-1 (PMC8448889; doi:10.1038/s41467-021-25802-1)
Supplement: Supplementary file 9 — Description of Additional Supplementary Files [file 41467_2021_25802_MOESM9_ESM.docx]

**Supplementary Movie 1.** Resistor network simulation of volatile metal-to-insulator resistive switching at the substrate temperature of 60 K.

**Supplementary Movie 2.** Resistor network simulations of volatile resistive switching due to the electrical triggering of a metal-to-insulator phase transition at the base temperature of 100 K.

**Supplementary Movie 3.** Resistor network simulation of volatile metal-to-insulator resistive switching at the substrate temperature of 150 K.

**Supplementary Movie 4.** Resistor network simulation of volatile metal-to-insulator resistive switching at the substrate temperature of 200 K.

**Supplementary Movie 5.** Resistor network simulation of volatile metal-to-insulator resistive switching at the substrate temperature of 250 K.

**Supplementary Movie 6.** Resistor network simulation of volatile metal-to-insulator resistive switching at the substrate temperature of 300 K.

**Supplementary Movie 7.** Resistor network simulation of volatile metal-to-insulator resistive switching at the substrate temperature of 330 K.
